# Epidemic Myalgia in Adults Associated with Human Parechovirus Type 3 Infection, Yamagata, Japan, 2008

**DOI:** 10.3201/eid1811.111570

**Published:** 2012-11

**Authors:** Katsumi Mizuta, Makoto Kuroda, Masayuki Kurimura, Yoshikazu Yahata, Tsuyoshi Sekizuka, Yoko Aoki, Tatsuya Ikeda, Chieko Abiko, Masahiro Noda, Hirokazu Kimura, Tetsuya Mizutani, Takeo Kato, Toru Kawanami, Tadayuki Ahiko

**Affiliations:** Yamagata Prefectural Institute of Public Health, Yamagata, Japan (K. Mizuta, Y. Aoki, T. Ikeda, C. Abiko, T. Ahiko);; National Institute of Infectious Diseases, Tokyo, Japan (M. Kuroda, T. Sekizuka, M. Noda, H. Kimura, T. Mizutani);; Yonezawa City Hospital, Yamagata (M. Kurimura, Y. Yahata);; and Yamagata University Faculty of Medicine, Yamagata (T. Kato, T. Kawanami)

**Keywords:** epidemic myalgia, human parechovirus, myositis, Japan, HPeV, parechovirus, Picornaviridae, viruses, adults, children, myalgia, HPeV3

## Abstract

Human parechovirus has rarely been shown to cause clinical disease in adults. During June–August 2008, a total of 22 adults sought treatment at Yonezawa City Hospital in Yamagata, Japan, for muscle pain and weakness of all limbs; most also had fever and sore throat. All patients received a clinical diagnosis of epidemic myalgia; clinical laboratory findings suggested an acute inflammatory process. Laboratory confirmation of infection with human parechovirus type 3 (HPeV3) was made for 14 patients; we isolated HPeV3 from 7 patients, detected HPeV3 genome in 11, and observed serologic confirmation of infection in 11. Although HPeV3 is typically associated with disease in young children, our results suggest that this outbreak of myalgia among adults was associated with HPeV3 infection. Clinical consideration should be given to HPeV3 not only in young children but also in adults when an outbreak occurs in the community.

## Abstract 2

This virus typically causes illness in young children but was found to be associated with illness in adults.

Human parechovirus (HPeV) is a positive-sense, single-stranded RNA virus belonging to the family *Picornaviridae* and the genus *Parechovirus* (*1*,*2*). HPeV type 1 (HPeV1) and HPeV2 were discovered in the United States in children with diarrhea in 1956; initially designated echovirus types 22 and 23, respectively, these viruses were recently reclassified and renamed (*1*,*2*). In 1999, HPeV3 was identified from a 1-year-old child with transient paralysis, fever, and diarrhea in Japan (*3*). Complete genome sequences are available for HPeV1–8, and viral protein (VP) 1 coding region sequences have recently been reported for HPeV9–16 (*1*,*4*).

HPeV1 and HPeV2 mainly cause mild gastrointestinal or respiratory illness, but more serious diseases have been occasionally reported, including myocarditis, encephalitis, pneumonia, meningitis, flaccid paralysis, Reye syndrome, and fatal neonatal infection (*2*,*5*,*6*). HPeV3 also causes not only mild gastrointestinal and respiratory tract illness but also severe illness in young children, including sepsis and conditions involving the central nervous system (*1*,*5*,*7*–*13*).

Although the seroprevalence of the recently discovered HPeV4–8 are unknown, HPeV1–3 infections usually occur in early infancy (*1*,*3*). Because all children have antibodies against HPeV1 after 1 year of age, HPeV1 seroconversion during the early months of life has been clearly established (*2*). HPeV3 is reported to infect younger children more often than HPeV1; HPeV3 infections occur most commonly among infants <3 months of age (*1*,*10*,*11*). In contrast, reports in the literature that describe HPeV3 infection in persons >10 years of age are rare (*1*,*14*).

An unusual outbreak of epidemic myalgia among adults occurred during June–August 2008 in Yonezawa, Yamagata, Japan, and epidemiologic investigation found that it was associated with HPeV3 infection. We describe this outbreak and provide expanded information about the clinical spectrum of HPeV3 infection.

## Methods

### Case-Patients

During June 17–August 6, 2008, an outbreak of an unknown disease was observed among adults in Yonezawa, Yamagata, Japan. A total of 22 patients who lived in the city of Yonezawa who had myalgia, muscular weakness, sore throat, and orchiodynia (among men) sought treatment at Yonezawa City Hospital. Because all had symptoms of severe myalgia, these patients were given a diagnosis of with acute myalgia syndrome of unknown cause. The patients consisted of 15 men and 7 women ages 25–66 years (mean 37 years). Because several patients had contact with other persons who had similar symptoms, the outbreak was considered to be associated with an infectious agent. Virologic and serologic analyses were carried out to find the associated agent. This study was approved by the Ethics Committee of the Yonezawa City Hospital.

### Screening for Pathogens

Throat swab and stool specimens were collected from 14 patients (Table 1). Throat swab specimens were placed immediately in tubes containing 3 mL of transport medium, and stool specimens were put in a stool container and transported to the Department of Microbiology, Yamagata Prefectural Institute of Public Health, Yamagata, to undergo virus isolation and reverse transcription PCR (RT-PCR) for enteroviruses, HPeV1, and HPeV2.

**Table 1:** Demographic and clinical data for 22 patients with epidemic myalgia, Yonezawa, Yamagata, Japan, June–August 2008*

Case-patient no.	Age, y/sex	Illness onset date	Hospital visit date	Clinical symptoms	Laboratory test results†			Virus isolation	RT-PCR for HPeV3		Neutralizing antibody titer against HPeV3 (d after illness onset)
					CPK	CRP	MYO		Throat swab/stool	Serum	
**1**	36/M	Jun 13	Jun 17	Myalgia, weakness, fever	222	2.3↑	66.0↑	Throat swab –, stool –	Throat swab –, **stool +**	–	**16 (21), 64 (61)**
2	66/F	Jun 16	Jun 17	Myalgia, weakness	66	1.5↑	26.0	ND‡	ND	–	64 (58)
3	25/M	Jun 20	Jun 25	Myalgia, weakness, fever, orchiodynia	201	0.8↑	148.0↑	ND	ND	–	16 (6), 16 (26)
**4**	31/M	Jun 21	Jun 24	Myalgia, weakness, fever, sore throat	239	0.5↑	66.0↑	ND	ND	**+**	**<8 (3), 16 (9), 16 (27)**
5	32/M	Jun 22	Jun 24	Myalgia, weakness, fever, sore throat	1,334↑	5.0↑	138.8↑	Throat swab –, stool –	Throat swab –, stool –	ND	ND
**6**	43/M	Jun 24	Jun 25	Myalgia, weakness, fever, sore throat	277	2.0↑	73.0↑	ND	ND	–	**<8 (3), 8 (6), 32 (64)**
**7**	30/F	Jun 26	Jul 2	Myalgia, weakness, fever,	581↑	0.1	110.0↑	ND	ND	–	**8 (6), 32 (15), 128 (71)**
**8**	28/M	Jun 29	Jul 1	Myalgia, weakness, fever, sore throat, orchiodynia	283	4.4↑	83.0↑	Throat swab –, **stool+**	**Throat swab +, stool+**	**+**	**<8 (2,3,5,8), 8 (17), 64 (59)**
**9**	39/F	Jun 30	Jul 7	Myalgia, weakness, fever, sore throat	262↑	0.7↑	81.0↑	Throat swab –, **stool +**	**Throat swab +, stool +**	–	**<8 (7), 8 (11), 32 (65)**
**10**	36/M	Jul 1	Jul 4	Myalgia, weakness, fever, sore throat	693↑	0.4↑	230.0↑	Throat swab –, **stool +**	Throat swab –, **stool +**	–	**8 (8), 64 (59)**
**11**	35/M	Jul 3	Jul 6	Myalgia, weakness, fever, sore throat	782↑	1.1↑	124.4↑	Throat swab –, **stool +**	Throat swab –, **stool +**	–	**<8 (3,4), 16 (6), 8 (22)**
**12**	38/M	Jul 3	Jul 6	Myalgia, weakness, fever, sore throat	321↑	0.2	201.0↑	Throat swab –, stool –	Throat swab –, stool –	–	**8 (3), 16 (4), 32 (13), 16 (57)**
**13**	38/F	Jul 4	Jul 4	Myalgia, weakness, fever, sore throat	166↑	0.9↑	121.6↑	**Throat swab +,** stool –	**Throat swab +, stool +**	–	**<8 (0,3,5), 16 (14), 256 (56)**
**14**	37/F	Jul 6	Jul 12	Myalgia, weakness, seizures	426↑	0.2	253.4↑	Throat swab –, **stool +**	**Throat swab +, stool +**	–	**8 (8), 16 (12), 32 (24), 64 (59)**
**15**	48/M	Jul 8	Jul 11	Myalgia, weakness, sore throat	1,048↑	1.9↑	193↑	Throat swab –, stool –	**Throat swab +, stool +**	ND	ND
16	41/M	Jul 9	Jul 11	Myalgia, fever, sore throat	88	6.8↑	29	Throat swab –, stool –	Throat swab –, stool –	–	8 (9,63)
17	26/F	Jul 11	Jul 14	Myalgia, weakness, fever,	45	0.7↑	18	Throat swab –, stool –	Throat swab –, stool –	–	16 (61)
**18**	36/M	Jul 11	Jul 14	Myalgia, weakness, fever, sore throat orchiodynia	355↑	0.9↑	87↑	Throat swab –, **stool +**	Throat swab ND, s**tool +**	–	8 (12)
19	23/M	Jul 17	Jul 18	Myalgia, weakness, fever, sore throat	165	1.7↑	31	Throat swab –, stool –	Throat swab ND, stool –	–	<8 (1)
20	34/M	Jul 19	Jul 20	Myalgia, weakness, fever,	1,598↑	0.5↑	75.2↑	ND	ND	–	16 (13,24)
21	38/F	Jul 31	Aug 4	Myalgia, weakness, fever, sore throat	43	5.5↑	35	ND	ND	–	<8 (4)
**22**	55/M	Aug 6	Aug 6	Myalgia, weakness, fever, sore throat, orchiodynia	556↑	4.9↑	26.8	ND	ND	**+**	<8 (1)

*Boldface indicates HPeV3 infection confirmation by virus isolation, RT-PCR, seroconversion, 4-fold increase of neutralizing antibody. HPeV3, human parechovirus type 3; RT-PCR, reverse transcription PCR; CPK, creatine phosphokinase; CRP, C-reactive protein; MYO, myoglobin; –, negative; +, positive; ND, not done. †Arrows indicate finding above reference levels: CPK, M: 60–287 IU/L, F: 45–163 IU/L; CRP, 0–0.3 mg/dL; MYO, M 60, F 35 ng/mL.

Virus isolation was carried out using a described microplate method (*15*). In brief,, HEF, HEp-2, Vero E6, MDCK, RD-18S, and GMK cell lines were prepared in the wells of a 96-well microplate (Greiner Bio-One, Frickenhausen, Germany). After a medium change, throat swabs and 10% stool suspension specimens were centrifuged at 1,500 × *g* for 20 min, and 75 μL of the supernatant was added to 2 wells of each of the cell lines. The inoculated plates were centrifuged for 20 min at 450 × *g*, incubated at 33°C in a 5% CO_2_ incubator, and assessed for cytopathic effect.

RNA was extracted from 200 μL of each throat swab specimen or 10% stool suspension using a High Pure Viral RNA Kit (Roche Diagnostics, Manheim, Germany) according to the manufacturer’s instructions and then transcribed into complementary DNA (cDNA) as described (*16*). PCR was performed as described (*17*,*18*), except that we used a mixture of 224 and 222 primers for the first PCR and a mixture of AN89 and AN88 primers for the nested PCR to detect enteroviruses and K28, K29, and K30 primers to detect HPeV1 and HPeV2. The remainder of each specimen and the cDNA specimens were stored at −80°C.

### Ultra-high Throughput Direct Sequencing Analysis

Ultra-high throughput direct sequencing analysis was carried out as described (*19*). Total DNA or RNA was prepared from the specimens of case-patient 11 by using a viral nucleic acid purification kit (Roche Diagnostics). Double-stranded cDNA (ds-cDNA) was prepared from 1 µg of total RNA using the random priming method with the SuperScript Choice System for cDNA synthesis (Invitrogen, Carlsbad, CA, USA). A mixture of DNA and ds-cDNA was purified by using a QIAquick PCR Purification kit (QIAGEN, Hilden, Germany).

An ≈300-bp length DNA library was prepared from a mixture of 2 µg of total DNA and ds-cDNA by using a genomic DNA sample prep kit (Illumina, San Diego, CA, USA), and DNA clusters were generated on a slide using a Single Read Cluster Generation kit version 4 on an Illumina cluster station (Illumina) according to the manufacturer’s instructions. To obtain ≈1.0 × 10^7^ clusters for 1 lane, the general procedure as described in the manufacturer’s standard recipe was performed. All sequencing runs for 83-mers were performed with GA II using the Illumina Sequencing Kit version 5. Fluorescent images were analyzed using the Illumina SCS2.8/RTA1.8 to obtain FASTQ formatted sequence data. The obtained DNA sequence reads were investigated by using a MEGABLAST search (*20*) with an e^–5^ e-value cutoff against the nonredundant nt database nt, followed by taxonomic classification using MEGAN version 3.9.0 (*21*) with the following parameters: minimum support 1; minimum score 35.0.

RT-PCR was performed by using ≈100 ng of total RNA, the appropriate primer pair, and the PrimeScript II High Fidelity One-Step RT-PCR Kit (TaKaRa, Shiga, Japan). The following quantitative RT-PCR program was used: reverse transcription reaction 45°C for 10 min; initial denaturation 94°C for 2 min; and 3 steps of amplification (× 35 cycles) at 98°C for 10 s, 55°C for 15 s, and 68°C for 1 min. PCR products were resolved and purified by agarose gel electrophoresis and then sequenced by Sanger sequencing by using a BigDye Terminator v3.1 Cycle Sequencing Kit (Applied Biosystems, Foster City, CA, USA).

### Repeat of Virus Isolation and Molecular Detection for HPeV3

After HPeV3 was detected in the specimens from case-patient 11 by ultra-high throughput direct sequencing analysis, to investigate whether HPeV3 was associated with the myalgia epidemic, we repeated the virus isolation focusing on HPeV3, using GMK, Vero, and LLC-MK2 cell lines using the 28 stocked throat swab and stool samples shown in Table 1. We used 2 LLC-MK2 cell lines, one provided by the National Institute of Infectious Diseases Japan and the other by the Niigata Prefectural Institute of Public Health and Environmental Sciences. We also attempted to specifically amplify the HPeV3 genome via RT-PCR using frozen cDNA and our original primers (Parecho3-VP1F1Y and Parecho3-VP1R1Y for the first PCR and Parecho3-VP1F2Y and Parecho3-VP1R2Y for the nested PCR [Table 2]), using the same conditions as in the preliminary screening procedure. When the RT-PCR result was positive, PCR products were purified and sequenced on a Sanger sequencer using a BigDye Terminator v3.1 Cycle Sequencing Kit (Applied Biosystems) and the primers listed in Table 2. We also attempted to amplify the HPeV3 genome using RT-PCR from all serum specimens that were initially collected for serologic analysis.

**Table 2:** Primers used to detect and sequence analysis for human parechovirus 3 among patients with epidemic myalgia, Yonezawa, Yamagata, Japan, June–August 2008

Primer	Nucleotide sequence, 5′ → 3′	Nucleotide position*
Parecho3-F2K	AACAAGTGACACTATGGATCTGATC	576–601
Parecho3-F3K	CAAAGTAGCAGATGATGCTTCCAA	824–849
Parecho3-F4K	CAAGCCAAATATTTTGCTGCAGTAA	1165–1190
Parecho3-F5K	TGCATTGGTGGTTTATGAGCCTAA	1244–1268
Parecho3-F21K	TCAGACAACACCACACCTTCAAG	1822–1844
Parecho3-VP1F1Y	GGGCCTTTGGGTAATGAGAAA	2452–2472
Parecho3-VP1F2Y	TGACAACATATTTGGTAGAGCTTGGT	2538–2563
Parecho3-F6K	AGGAGATAATGTATATCAATTGGAT	3095–3120
Parecho3-F7K	TGACGGCTGGTTTAATGTCAACTAT	3368–3392
Parecho3-F8K	CTGAATCAATGTCCAACACAGACGA	3737–3761
Parecho3-F9K	TGGACTATGCCTCTGATATTATTGT	3994–4019
Parecho3-F10K	AATAATGGCCATTTGCTTTAGGAGT	4098–4122
Parecho3-F11K	CTTGTAAATTAAATGGTGTGTACAC	4304–4328
Parecho3-F12K	ACTAGGAAGGAGAAAGATATTGAAA	4402–4426
Parecho3-F13K	CAGCCAAAGCATATAGTAGGGCTG	4609–4633
Parecho3-F14K	TTGAACAAATGGAAGCCTTCATTGA	4916–4940
Parecho3-F15K	GTTGTAGACTGGTTCAGTAGTAAG	5011–5034
Parecho3-F16K	AAAGGAACTTTCCCAGTCACGCAGA	5201–5225
Parecho3-F17K	CAGAGAGTATGTTGATTTGGATGAC	5553–5576
Parecho3-F18K	GTGGCTATTCCTTTCAATTTTCTT	5778–5802
Parecho3-F19K	GGCCCAGCAGTTTTAAGCAAATCAG	5863–5947
Parecho3-F20K	TTGTCATGATTCACCTGATCTTGTC	6608–6633
Parecho3-R13K	AGTTTGTGGTATTTACAGTGGTTGT	912–938
Parecho3-R11K	TTTTAACGTAGTTTGTGTCTGCA	1380–1402
Parecho3-R15	CATGTATAGAATATGAATGTTTATT	2673–2697
Parecho3-VP1R2Y	ACCCCTGCTCTGCCATGTATA	2693–2710
Parecho3-VP1R1Y	TCCCGTGCATCATTGGTCTA	2883–2902
Parecho3-R10K	TGACAGATGATTCAAGATACTTCAC	3238–3253
Parecho3-R9K	AGTGGTACACTTCTGCACAAGTAAG	3468–3492
Parecho3-R8K	ATCCACCAATCAATATGTCTGAATG	3859–3884
Parecho3-R7K	TGGATAGTGTGTGTGTTAGGAAAGA	4264–4288
Parecho3-R5K	CCACTTTAGAAATAAGCAGACCACC	5701–5725
Parecho3-R4K	CCATTTTGACTTCCACTGCTCCA	5901–5923
Parecho3-R3K	CCTAAATTTGGACTTGACACAGG	5993–6015
HPeV3whole-RT-RK	TTTTGGTATGTCCAATATTCCAAATTAGTG	7296–7321

*Relative to human parechovirus 3 strain A308/99 (GenBank accession no. AB084913).

### Serologic Study

To further evaluate the role of HPeV3 in this outbreak of myalgia, we measured the neutralizing antibody response against HPeV3 in 1–5 serum samples from 20 of the patients (Table 1). To observe seroconversion and changes in antibody titers, we measured neutralization antibodies using a microneutralization test on a 96-well microplate. Sample serum samples were inactivated at 56°C for 30 min and then diluted from 1:8 to 1:4,096 by serial 2-fold dilution. One HPeV3 isolate in this outbreak in Yamagata (1356-Yamagata-2008) was used as a challenge virus antigen. We mixed and incubated (37°C, 60 min) 60 μL of each diluted serum sample with 60 μL of virus fluid containing ≈10^2^ 50% tissue culture infectious dose. We prepared confluent monolayers of the LLC-MK2 cell line provided by the Niigata Prefectural Institute of Public Health and Environmental Sciences, washed the cells with phosphate-buffered saline without calcium or magnesium, added 50 μL of maintenance media, and injected 50 μL of each incubated virus–serum mixture into each of 2 cultures. The plates were then incubated in a 5% CO_2_ incubator at 33°C for cytopathic effect observation. The reciprocal value of the highest dilution of serum neutralizing the virus compared to the control was taken to be the titer. Seropositivity was defined as titer >8. Virus infection was considered confirmed if seroconversion from negative to positive could be documented. Alternatively, if all serum samples were positive, a 4-fold increase in antibody titer was considered serologic evidence of infection with HPeV3.

## Results

### Case-Patients

The 22 case-patients in this outbreak had similar symptoms (Table 1); all had severe myalgia involving mainly the proximal muscles of the upper and lower extremities. The next most common symptom was muscular weakness in the arms and legs (21, 95.5%), followed by fever (19, 86.4%), pharyngitis (15, 68.2%), orchiodynia (4, 18.2%), and seizures (1, 4.5%). Symptoms worsened rapidly within 1–2 days after onset. Eight patients were hospitalized on a visit to Yonezawa City Hospital and remained hospitalized for 5 (4 patients), 6 (1 patient), 8 (2 patients), or 10 days (1 patient); mean hospitalization was 6.5 days. Case-patient 14 had seizures and subsequent disturbance of consciousness. For all patients, muscular weakness improved as muscle pain decreased.

### Laboratory Findings

Creatinine phosphokinase (CPK), C-reactive protein, and myoglobin levels were higher than reference ranges in 12, 19, and 16 patients, respectively (Table 1). Elevated CPK levels returned to normal within several days. An examination of cerebrospinal fluid showed no sign of inflammation.

Magnetic resonance imaging (MRI) of the muscles of case-patients 4, 13, and 15 showed increased signal intensity on T2-weighted images. We suspected that case-patient 14 had encephalitis on the basis of clinical symptom, but results of MRI of the brain and cerebrospinal fluid examination did not support this diagnosis. Clinical symptoms and laboratory findings for all patients were consistent with inflammatory myositis.

### Detection of Potential Pathogens

No viruses were detected by screening steps at the Yamagata Prefectural Institute of Public Health using virus isolation and RT-PCR targeting enteroviruses, HPeV1, and HPeV2. Thus, we investigated possible uncharacterized pathogens by using direct DNA sequencing. To determine potential pathogens for the patients, we performed direct sequencing of a mixture of purified DNA and ds-cDNA from the total RNA extracted from either the throat swab or stool specimens of case-patient 11. No other possible viral sequences were detected by the high-throughput sequencing for case-patient 11.

Next-generation DNA sequencer GA II produced ≈1.5 × 10^7^ 83-mer short reads from the mixed DNA library. To exclude the human-derived read sequences, all obtained reads were initially aligned to a reference sequence of human genomic DNA, followed by quality trimming to remove low-quality reads and exclude reads with similarities to ambiguous human sequences. All remaining possible pathogen reads were further analyzed using a MegaBLAST search against nonredundant databases.

One type of HPeV was found in the analyzed specimens. Three HPeV reads were identified from the throat swab specimen and 1,505 HPeV reads from the stool specimen of case-patient 11. To further characterize the type of HPeVs detected, de novo assembly was performed by using Euler-SR version 1.0 (*22*); the resulting partial contigs showed higher similarity to HPeV3 than to other HPeVs.

**Figure 1:**
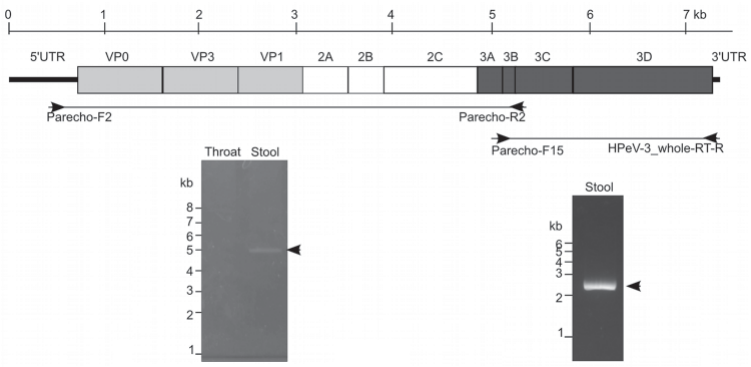
Schematic representation of the human parechovirus 3 (HPeV3) genome sequence and coding polyprotein. Reverse transcription PCR results are shown below the sequence. VP, viral protein; UTR, untranslated region.

**Figure 2:**
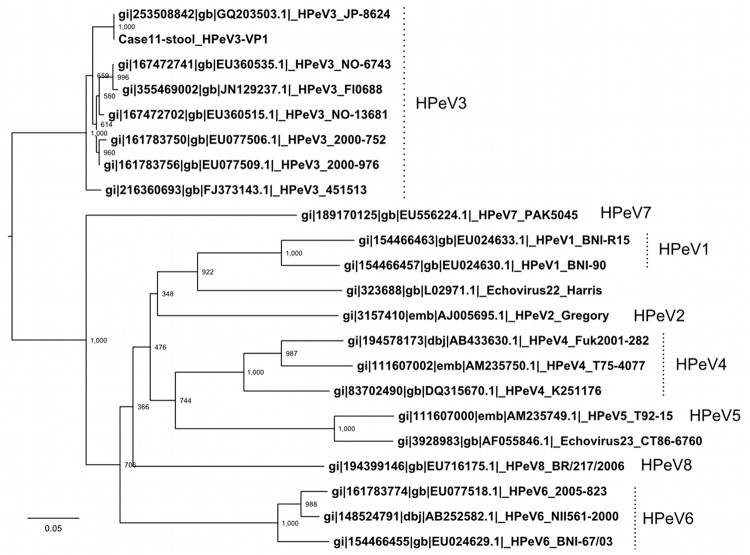
Phylogenetic tree of the viral protein 1 region sequence in the available human parechovirus (HPeV) genomes, including HPeV1–8. The tree was constructed by the neighbor-joining method with 1,000× bootstrapping. Scale bar indicates nucleotide substitutions per site.

To determine the whole HPeV sequence for the isolates we obtained, RT-PCR was performed (Figure 1), and the products were sequenced. The whole coding nucleotide sequence of the polyprotein in the HPeV detected in the stool sample from case-patient 11 was aligned against all available HPeV complete genomes, including HPeV1–8. A phylogenetic tree was generated for the VP1 region (Figure 2), and the HPeV in the stool specimen of case-patient 11 was identified as HPeV3. All detected HPeV3 VP1 sequences among the 2008 myalgia cases were identical (Table 1), except that we found a single nucleotide substitution in sequences from the serum samples from case-patients 4 and 22.

### Repeat of Virus Isolation and Molecular Detection Targeting HPeV3

We passaged isolates 6 times using GMK and LLC-MK2 cell lines, but all strains except 1 (isolated after 5 passages) were recovered within 3–4 passages. We could not isolate HPeV3 using the LLC-MK2 cell line provided by the National Institute of Infectious Diseases Japan, but we were successful when using LLC-MK2 cell line from the Niigata Prefectural Institute of Public Health and Environmental Sciences. In total, we isolated HPeV3 strains from either the throat swab or stool specimen of 7/14 patients analyzed (Table 1).

RT-PCR was successful in detecting the HPeV3 genome in the throat swabs or stool specimens from 9/14 patients (Table 1). We also detected the HPeV3 genome in 3 serum specimens collected within 3 days after the onset of illness (Table 1). Sequence data were registered under GenBank accession nos. AB668029–AB668045.

### Serologic Study

Of 20 analyzed patients, seroconversion was observed in 6 patients. A 4-fold increase in neutralization antibodies against HPeV3 was confirmed in 5 patients (Table 1).

## Discussion

By conducting virus isolation, RT-PCR, and serologic examination, we confirmed an outbreak of epidemic myalgia among adults in Yonezawa, Yamagata, Japan, during June–August 2008 was associated with HPeV3 infection. Although we did not detect any virus in our first screening, direct sequencing analysis suggested that these patients were infected with HPeV3. We next tried to detect this virus specifically and isolated HPeV3 strains from the throat swabs or stool specimens of 7 patients; detected the virus genome in the throat swabs or stool or serum specimens of 11 patients; and observed seroconversion or 4-fold increases in antibodies against HPeV3 in 11 patients. Altogether, we confirmed HPeV3 infection in 14 of the 22 patients in this outbreak. All patients, except 1 who experienced complications related to epilepsy, recovered completely within 1 week after the onset of illness through treatment with antiinflammatory drugs only.

Enteroviruses have been implicated in the pathogenesis of human neuromuscular diseases because of their association with certain acute and chronic acquired myopathies and paralytic motor neuron syndromes (*23*). Epidemic pleurodynia (Bornholm disease), an acute febrile illness with myalgia caused by picornaviruses such as group B coxsackieviruses, is perhaps best known (*24*–*26*). However, in this outbreak, none of the patients showed the chest and abdominal pain typical of pleurodynia; they instead showed muscular weakness, which is not generally observed in epidemic pleurodynia (*23*,*25*). Conversely, it has been reported that patients with acute enterovirus myositis experience fever, chills, myalgia, and generalized weakness and that thigh muscle or generalized muscle involvement may occur (*25*,*26*). Among these patients, laboratory studies may demonstrate myoglobinemia, myoglobinuria, and an elevated levels of muscle enzymes such as CPK (*25*,*26*). The HPeV3 outbreak in Yonezawa resembles these descriptions of acute myositis outbreaks; patients commonly had fever, myalgia, and muscle weakness mainly in the arms and legs, and laboratory findings and MRI studies suggested the presence acute inflammation around the peripheral muscles. Thus, we concluded that the disease in this outbreak was an acute inflammatory muscle disease associated with HPeV3 infection in adults.

In enterovirus viremia, viruses enter through the oral or respiratory route, replicate in the pharynx and alimentary tract, spread to multiple organs such as the central nervous system, heart, and skin, and then diminish and disappear after neutralizing serum antibodies are produced (*24*). HPeV3 has been reported to cause severe systemic diseases, especially in neonates and infants (*1*,*7*,*11*,*13*). HPeV3 has been isolated using the Vero cell line or detected by real-time PCR in not only nasopharyngeal swabs but also in the lungs, colons, and spleens of dead infants (*27*).

In this study, we showed HPeV3 viremia in 3 patients without neutralizing antibodies within a few days after the onset of illness. Thus, we conclude that HPeV3 viremia affects many organs, including the peripheral muscles as well as the organs normally targeted by enteroviruses. Several male patients in the Yonezawa outbreak also had orchitis, which is described as a symptom of epidemic pleurodynia (*24*) but may also be associated with systemic infection, although we have no evidence to support this hypothesis.

HPeV often grows poorly in culture, and typing reagents are not widely available for newly discovered types (HPeV3–14) (*1*,*28*). In particular, HPeV3 is difficult to culture in standard diagnostic cell lines, and its isolation is largely determined by the cell lines used (*14*). So far, Vero, LLC-MK2, Caco2, and BSC-1 cell lines have been used (*3*,*7*,*8*,*10*,*14*,*27*). In the first screening in our study, we could not isolate HPeV3 using 6 cell lines that we use routinely (*15*). However, we succeeded in whole-sequence analysis using a stool specimen from case-patient 11, which suggests the failure to isolate HPeV3 was not a result of low viral load in the specimens from the patients with epidemic myalgia. In our second attempt at isolation using several monkey kidney cell lines, such as GMK and LLC-MK2, we were able to isolate HPeV3 after only several passages. Although 2 LLC-MK2 cell lines were used for HPeV3 isolation, the susceptibility of the lines to HPeV3 infection differed, further demonstrating the difficulties in culturing HPeV3. Because we analyze range of respiratory viruses, which are isolated from nasopharyngeal specimens, we routinely culture specimen-inoculated plates at 33°C; however, to isolate HPeV3, plates were cultured at 37°C.

Although we used a new direct-sequence analysis method and a slightly older RT-PCR method to amplify the HPeV3 genome in this study, new laboratory diagnostic procedures for HPeVs have been developed during the past few years. HPeV RT-PCRs now exist to detect all known HPeV types with great sensitivity (*29*), and HPeV3 and all the other HPeVs can be typed by nested or semi-nested PCR coupled with sequencing of the VP1 gene (*30*).

Our finding of HPeV3 infection in adults conflicts with most of the literature, which suggests that HPeV3 infections occur in early infancy (*1*,*3*,*7*,*10*,*11*,*13*). Harvala et al. observed that HPeV3 infections were seen exclusively in children <3 months of age (*1*,*11*,*13*). Watanabe et al. reported that HPeV3 was isolated from a 35-year-old woman with influenza-like illness, while 12/16 HPeV3-isolated cases were from patients <3 years of age (*14*). Our findings show that epidemic myalgia associated with HPeV3 infection might occur among adults, particular among those ≈30–40 years of age.

It remains unclear why these HPeV3 infections occurred among adults in 2008. HPeV3 infections occur in the spring and summer seasons, whereas HPeV1 infections are observed in small numbers throughout the year but predominantly during the fall and winter (*9*,*10*,*12*,*31*). National surveillance data for infectious diseases in Japan show HPeV3 yearly detection numbers was 1, 2, 52, 1, and 81 cases each year for 2004–2008; 117 cases (85.4%) were detected during June–September (*32*). The higher number of cases in 2008, coupled with a reported epidemic of HPeV3 among children, most <3 months of age, in Hiroshima Prefecture, Japan (*33*), indicates a possible summer outbreak of HPeV3 in 2008 in Japan. We postulate that an outbreak of HPeV3 among children was a necessary background condition for the outbreak of epidemic myalgia among adults in 2008.

In conclusion, we document detection of HPeV3 infection among adult patients with epidemic myalgia in Yamagata, Japan, during 2008. Clinical consideration should be given to HPeV3 not only in young children but also in adults when an HPeV3 outbreak occurs in the community. Continued research on adults with HPeV3 infection is needed to further understand the etiology and epidemiology of HPeV3.
